# Not Your Average Seizure: A Case of N-Methyl-D-Aspartate Receptor Encephalitis and Review of Literature

**DOI:** 10.7759/cureus.9068

**Published:** 2020-07-08

**Authors:** Talal Alkayali, Stephen Bell, Caitlin Bass, Natalia Lattanzio, Natan Kraitman

**Affiliations:** 1 Internal Medicine, Sarasota Memorial Hospital, Florida State University College of Medicine, Sarasota, USA

**Keywords:** autoimmune encephalitis, ovarian teratoma, seizure, anti-n-methyl-d-aspartate

## Abstract

Encephalitis is an inflammatory process of the brain that is most commonly related to infectious etiology; nonetheless, autoimmune encephalitis has been an increasingly identified entity that can cause it as well and should be considered. N-methyl-D-aspartate (NMDA) receptor encephalitis is a recently identified process but remains less recognized than autoimmune encephalitis. We report a case of an 18-year old female who initially presented with seizures and later developed behavioral symptoms of agitation, crying, screaming, and emotional lability. Ultimately, she was found to have NMDA receptor encephalitis related to ovarian paraneoplastic teratoma. The patient was treated with anti-epileptics and intravenous immunoglobulin and underwent oophorectomy that lead to her recovery. This case highlights the importance of early recognition of NMDA receptor encephalitis to facilitate appropriate investigations and management.

## Introduction

A clinical presentation of encephalitis is most often considered to be an infectious etiology. However, an often underdiagnosed and treatable cause is N-methyl-D-aspartate (NMDA) receptor encephalitis. This condition is part of a rare group of disorders referred to as autoimmune encephalitides. Presentations vary from limbic encephalitis to syndromes with complex neuropsychiatric symptoms such as deficits in memory, cognition, psychosis, seizure, abnormal movements, or coma. Frequent dyskinesias, such as opisthotonos and choreoathetoid movements as well as language impairment, may also be seen [1-2]. Wandinger et al. describe a prodromal phase characterized by flu-like symptoms followed by a psychotic phase characterized by behavioral changes and psychosis and the advanced stage characterized by decreased consciousness, seizures, and autonomic dysregulation [2]. Heightened suspicion is necessary to make this diagnosis due to a paucity of cases.

The most well described but still underrecognized etiology of autoimmune encephalitis is NMDA receptor encephalitis. NMDA receptors are glutamate receptors and ion channel proteins located in the post-synaptic membranes; these receptors serve as ligand-gated cation channels, with major significance for synaptic transmission and plasticity, especially the glutamate receptors subunit (GluN). When cerebrospinal fluid (CSF) immunoglobulin (Ig) G antibodies (Abs) form against the GluN1 subunit of the NMDA receptor, it leads to the development of anti-NMDA receptor encephalitis [2]. We present a case of anti-NMDA receptor encephalitis presenting as new-onset seizures in an 18-year-old female.

## Case presentation

An 18-year-old female without a past medical history presented to the emergency department complaining of headache and seizure-like activity for two days. She presented with a throbbing, occipital headache, with associated photophobia and phonophobia. Her family reported multiple episodes of staring into space, repeated abnormal jaw movements, and urinary incontinence. She had one witnessed tonic-clonic seizure lasting three minutes, which spontaneously aborted and was followed by a postictal state for 15 minutes. She denied fever, chills, motor weakness, blurry vision, or any other symptoms. She had no sick contacts and recent travel, and did not take any medications including herbal supplements, but she did have unprotected sexual intercourse two weeks prior and took “morning-after pill” levonorgestrel. She denied alcohol and illicit drug use. The patient is a high school student who works part-time in a convenience store after school.

Physical examination showed an alert and oriented female with normal vital signs and in no acute distress, with cranial nerves intact, normal speech, normal motor, and sensation in all extremities, normal deep tendon reflexes, and no ataxia. There were bite marks and erythema noted in the right cheek mucosa; otherwise, the examination was normal.

Initial work-up showed normal complete blood count, electrolytes, blood glucose, liver function, negative urine drug screen, normal brain magnetic resonance imaging (MRI) with/without contrast, and normal 30-minute electroencephalogram (EEG) with no seizure activity.

Two days into her hospital stay, she was noted to again have multiple staring episodes followed by confusion, as well as new behavioral symptoms with screaming, tearing, agitation, and difficulty with words finding. A continuous EEG was performed and found multiple seizures, arising from the left hemisphere and spreading to the right frontal region. These seizures lasted between 20 seconds to two minutes, and during seizure activity she was noted to have an elevation of her right arm, with facial grimacing or smiling, and at times left-sided head turn with unresponsiveness.

Lumbar puncture (LP) was performed, and CSF results are outlined in Table 1.

**Table 1:** Cerebrospinal fluid analysis RBCs, red blood cells; WBCs, white blood cells

	Results	Normal values
Appearance	Clear/colorless	Clear/colorless
RBCs	0	0-5 cells/uL
WBCs	106	0-5 cells/uL
Polymorphonucleocytes	1%	0-5%
Monocytes	99%	5-100%
Protein	28	15-45 mg/dL
Glucose	65	40-75 mg/dL

A BioFire® FilmArray meningoencephalitis panel testing 14 common bacterial, viral, and fungal pathogens by virtue of polymerase chain reaction (PCR) on a CSF specimen returned negative for Neisseria meningitides, Streptococcus pneumonia, Streptococcus agalactiae, Listeria monocytogenes, Haemophilus influenza, Escherichia coli, Cytomegalovirus, Enterovirus, Herpes simplex virus [HSV] I and II, Herpesvirus 6, Parechovirus, Varicella zoster virus, and Cryptococcus neoformans). Additional CSF testing included nonreactive venereal disease research laboratory (VDRL) antibody test and West Nile virus IgM and IgG, and cultures for fluid, fungal, acid-fast bacilli were negative. No malignant cells were detected on cytology in the CSF specimen.

Given the initial negative LP with no infectious etiology, further evaluation was pursued with autoimmune studies and abdominal imaging due to suspicion of NMDA receptor encephalitis.

Autoimmune work-up from CSF was negative for glutamic acid decarboxylase (GAD65) Ab, α-amino-3-hydroxy-5-methyl-4-isoxazolepropionic acid receptor (AMPA-R) Ab, gamma-aminobutyric acid (GABA) B receptor Ab, contactin-associated protein 2 (CASPR2) IgG, leucine-rich glioma-inactivated 1 (LGI1) IgG, amphiphysin Ab, anti-glial/neuronal nuclear Ab 1 (AGNA-1), and anti-neuronal nuclear Ab (ANNA) 1, ANNA-2, and ANNA-3.

CSF was positive for anti-NMDA receptor 1 (anti-NR1) Abs at a titer of 1:32 (normal: <1:2), consistent with NMDA receptor encephalitis.

Further blood work-up revealed an elevated erythrocyte sedimentation rate of 64 mm/hour (normal: 0-20 mm/hour) and C-reactive protein of 5.3 mg/dL (normal: <0.3 mg/dL); however, it was negative for antinuclear Ab, antineutrophil cytoplasmic Ab vasculitis, human immunodeficiency virus (HIV), syphilis, and West Nile IgG/IgM.

**Figure 1:**
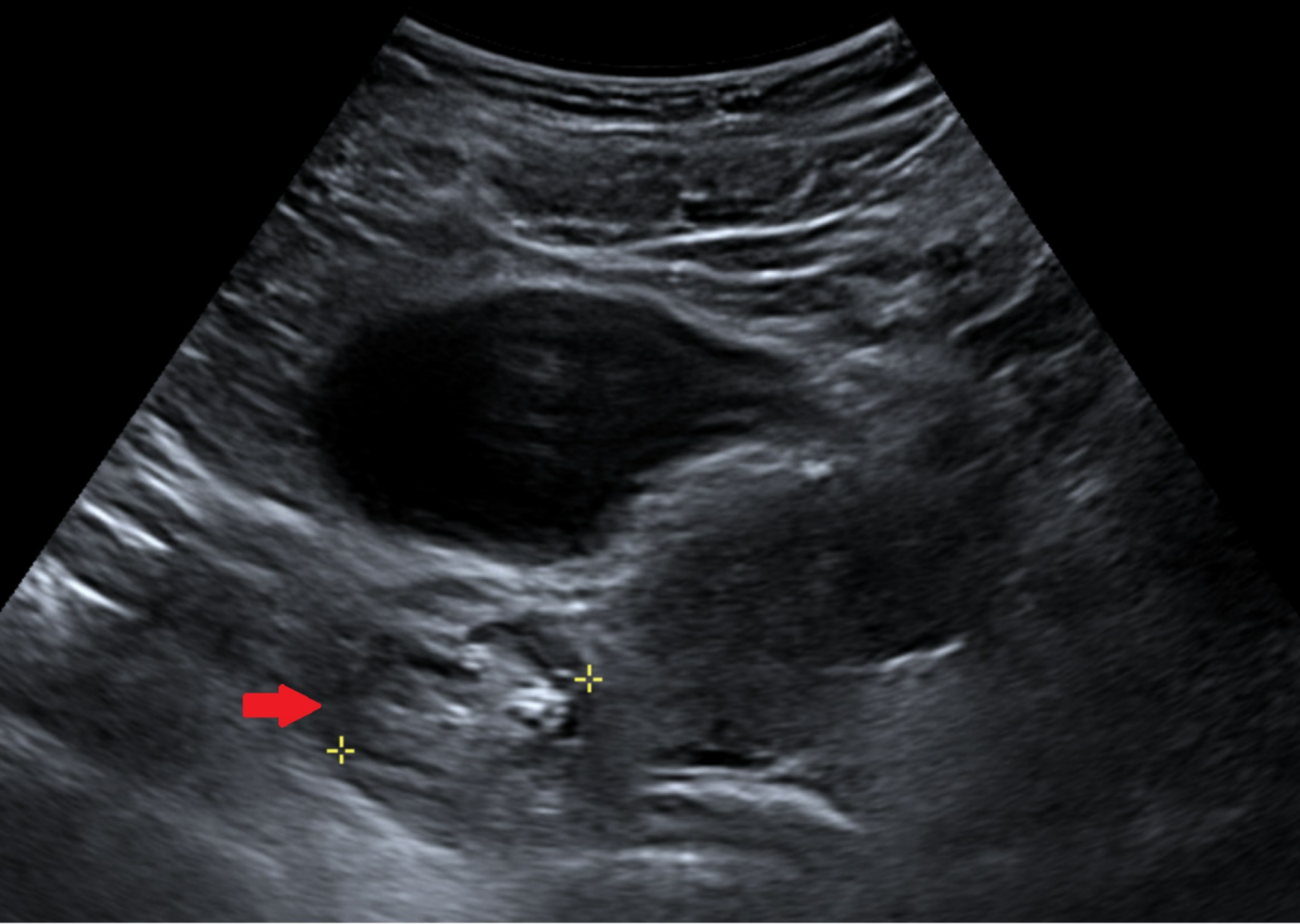
Transabdominal pelvic ultrasound Red arrow showing a solid mass in the right ovary.

While awaiting LP results, transabdominal pelvic ultrasound and pelvis MRI were obtained to look for underlying ovarian mass. Ultrasound results showed a solid lesion in the right ovary (Figure 1).

**Figure 2:**
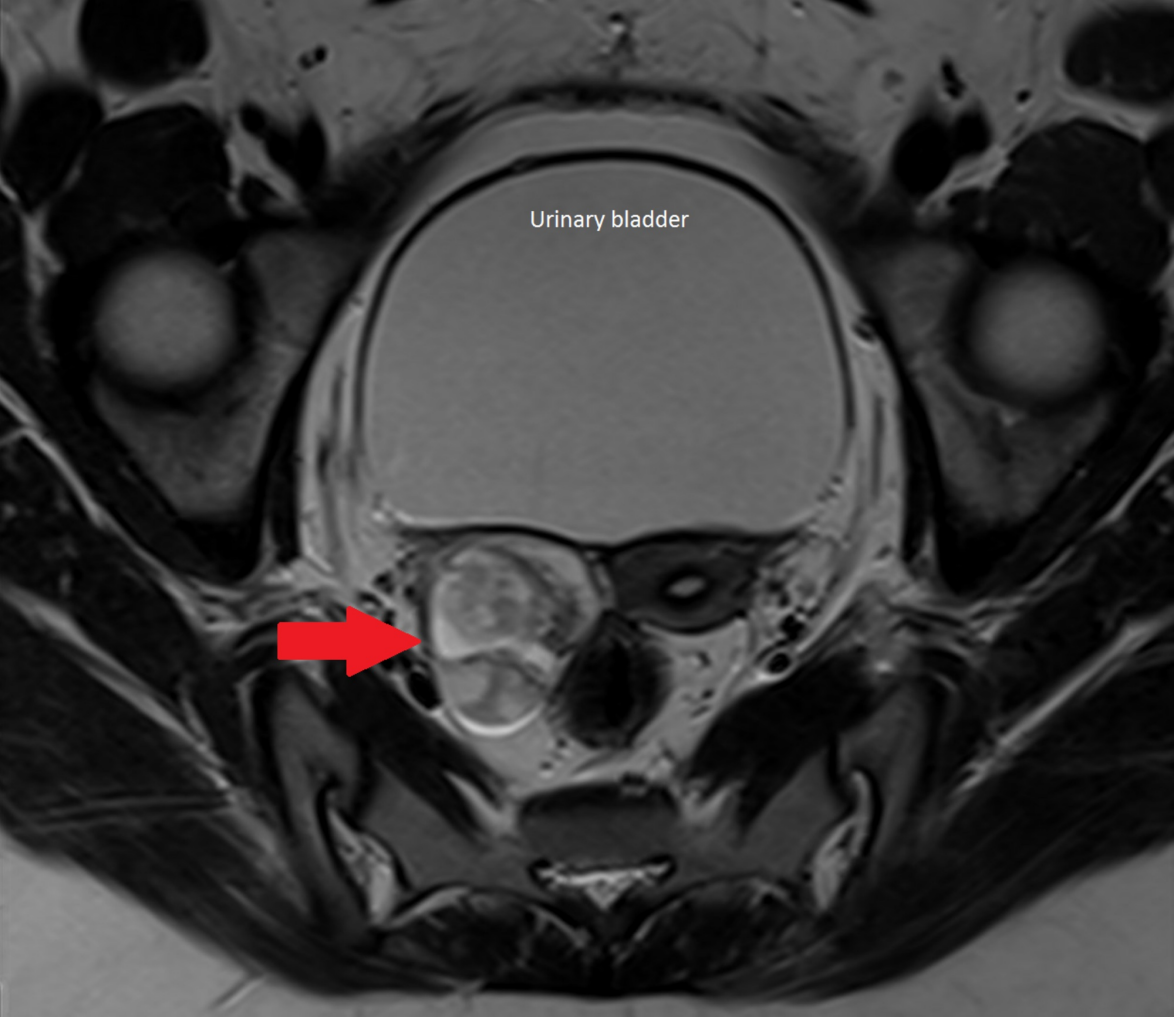
Pelvic MRI Red arrow showing a heterogeneous, complex, mixed solid, and cystic lesion in the right ovary.

MRI results showed a complex 2.9 x 3.7 x 2.5 cm mixed solid and cystic mass in the right ovary, demonstrating septal and nodular enhancement suspicious for teratoma (Figure 2).

Her alpha-fetoprotein level was 7 ng/mL (normal: <6.1 ng/mL). The patient was started on levetiracetam and then subsequently switched to fosphenytoin and valproic acid due to worsened agitation while on the levetiracetam. This combination controlled her seizures for a few days, but seizures restarted with worsening agitation and the patient became less interactive and non-verbal compared with her admission day. Fosphenytoin was continued, but valproic acid was switched to lacosamide and quetiapine added.

Methylprednisolone 80 mg intravenous every eight hours was started and was then increased to 500 mg every 12 hours for five days. Intravenous immunoglobulin (IVIG) 30 g daily for five days was initiated while finalizing LP results due to high suspicion for autoimmune encephalitis. Lamotrigine was added due to increased agitation.

The patient underwent right ovarian resection, with pathology showing high-grade immature teratoma (grade 3). After the surgery, no more seizure activity was reported, and significant improvement in mentation was noted within one week. Lamotrigine and fosphenytoin were stopped. The patient had ongoing episodes of agitation while being hospitalized that responded to repeat IVIG treatments 30 g daily for four to five days; she received a total of 14 IVIG treatments over a period of six weeks.

The patient was started on chemotherapy with etoposide and cisplatin for her grade 3 immature teratoma (T1cN0M0). She was discharged on lacosamide, quetiapine, and prednisone taper.

## Discussion

The diagnosis of NMDA receptor encephalitis can be challenging. Oftentimes, nonspecific and predominant neuropsychiatric symptoms may lead to misdiagnosis of a psychiatric disorder [3]. Neuropsychiatric sequelae are a potential problem due to delay in diagnosis as most patients are treated for infectious etiologies [4]. The majority of patients initially present with psychiatric symptoms at disease onset. Nearly eighty percent of cases are seen in women, of which half the cases occurring in women above the age of 18 are associated with a malignancy, most notably ovarian teratoma [5]. It is hypothesized that molecular mimicry may play a role in the development of this disease. Neural tissue in teratomas may share similar epitopes with the NR1 subunit of the NMDA receptor, leading to cross-reactivity of anti-tumor Abs.

Graus et al. propose the following criteria be met to diagnose autoimmune encephalitis: (i) subacute onset with rapid progression of less than three months of memory deficits, altered mental status, or psychiatric symptoms; (ii) at least one of the following: new focal central nervous system findings, new-onset seizures in the absence of a history of epilepsy, CSF pleocytosis, or MRI features of encephalitis; and (iii) exclusion of alternative diagnoses [6].

Radiology has not been shown to be helpful in making the diagnosis. However, decreased 18F-fluorodeoxyglucose (FDG) occipital lobe metabolism on positron emission tomography (PET) or brain computed tomography (CT) may be suggestive of limbic encephalitis [7]. In some cases, EEG can also support the diagnosis. Delta brush is a pattern characterized by a background of delta waves with superimposed bursts of beta frequency waves. While this is a uniquely recognized EEG pattern, it is neither sensitive nor specific for NMDA receptor encephalitis [8].

A definitive diagnosis is made with the detection of IgG Abs in the GluN1 subunit of the NMDA receptor in the serum or CSF. CSF IgG Ab is both highly sensitive and highly specific, whereas false-positive and false-negative results may occur with only serum testing [9].

Autoimmune encephalitides have been shown to be highly responsive to immunomodulatory therapy and, if present, tumor-directed therapy. Early initiation of treatment has been shown to improve clinical outcomes. Commonly used treatment strategies include high-dose corticosteroids, IVIG, plasma exchange, rituximab, cyclophosphamide, and mycophenolate, among others [10-11]. Bortezomib has been used in cases refractory to first- and second-line therapies with favorable results [11].

NMDA receptor encephalitis associated with a tumor generally has a favorable prognosis with tumor resection. Conversely, cases not associated with a tumor are more likely to have disease relapse [12]. Early diagnosis and treatment also confer a favorable prognosis. In patients not responding to first-line therapy, early initiation of therapeutic plasma exchange has been shown to improve clinical outcomes [13].

## Conclusions

The presentation of this patient is typical of NMDA receptor encephalitis. In our case, a young woman presented with psychiatric symptoms, but her infectious work-up was negative and a teratoma was found. The treatment response, however, was rather unusual. The patient showed minimal improvement with corticosteroids and IVIG, which is the treatment of choice. There was resolution of breakthrough seizures and an overall improvement of symptoms after oophorectomy. NMDA encephalitis is important in the differential diagnosis of patients presenting with encephalitis.
